# Direct Lentiviral-Cyclooxygenase 2 Application to the Tendon-Bone Interface Promotes Osteointegration and Enhances Return of the Pull-Out Tensile Strength of the Tendon Graft in a Rat Model of Biceps Tenodesis

**DOI:** 10.1371/journal.pone.0098004

**Published:** 2014-05-21

**Authors:** Charles H. Rundle, Shin-Tai Chen, Michael J. Coen, Jon E. Wergedal, Virginia Stiffel, Kin-Hing William Lau

**Affiliations:** 1 Musculoskeletal Disease Center, J. L. Pettis Memorial VA Medical Center, Loma Linda, California, United States of America; 2 Department of Medicine, Loma Linda University School of Medicine, Loma Linda, California, United State of America; 3 Department of Biochemistry, Loma Linda University School of Medicine, Loma Linda, California, United State of America; 4 Department of Orthopedic Surgery, Loma Linda University School of Medicine, Loma Linda, California, United State of America; The University of Hong Kong, Hong Kong

## Abstract

This study sought to determine if direct application of the lentiviral (LV)-cyclooxygenase 2 (*COX2*) vector to the tendon-bone interface would promote osteointegration of the tendon graft in a rat model of biceps tenodesis. The LV-*COX2* gene transfer strategy was chosen for investigation because a similar *COX2* gene transfer strategy promoted bony bridging of the fracture gap during bone repair, which involves similar histologic transitions that occur in osteointegration. Briefly, a 1.14-mm diameter tunnel was drilled in the mid-groove of the humerus of adult Fischer 344 rats. The LV-*COX2* or *βgal* control vector was applied directly into the bone tunnel and onto the end of the tendon graft, which was then pulled into the bone tunnel. A poly-L-lactide pin was press-fitted into the tunnel as interference fixation. Animals were sacrificed at 3, 5, or 8 weeks for histology analysis of osteointegration. The LV-*COX2* gene transfer strategy enhanced neo-chondrogenesis at the tendon-bone interface but with only marginal effect on *de novo* bone formation. The tendon-bone interface of the LV-*COX2*-treated tenodesis showed the well-defined tendon-to-fibrocartilage-to-bone histologic transitions that are indicative of osteointegration of the tendon graft. The LV-*COX2 in vivo* gene transfer strategy also significantly enhanced angiogenesis at the tendon-bone interface. To determine if the increased osteointegration was translated into an improved pull-out mechanical strength property, the pull-out tensile strength of the LV-*COX2*-treated tendon grafts was determined with a pull-out mechanical testing assay. The LV-*COX2* strategy yielded a significant improvement in the return of the pull-out strength of the tendon graft after 8 weeks. In conclusion, the *COX2*-based *in vivo* gene transfer strategy enhanced angiogenesis, osteointegration and improved return of the pull-out strength of the tendon graft. Thus, this strategy has great potential to be developed into an effective therapy to promote tendon-to-bone healing after tenodesis or related surgeries.

## Introduction

Lesions of the long head of biceps tendons are often disabling because of pain, weakness, and/or debilitating pseudoparalysis of the shoulder that lead to poor sleep quality and decreased ability to independently perform daily activities [1]. Treatment options depend on patient's age, co-morbidities, activity level, and extent of the disability [2]. Conservative treatment with modalities, e.g., nonsteroidal anti-inflammatory drugs (NSAIDs), corticosteroid injections, gentle physical therapy, and periods of rest, is attempted initially [3]. If symptoms persist, surgical intervention is indicated. Of the two major surgical options (tenotomy and tenodesis) [4], biceps tenodesis continues to be a popular surgical option for younger, physically active, and motivated patients.

Biceps tenodesis is a surgical procedure that releases the injured tendon from its attachment into the labrum and anatomically reattaches it to the humerus in the bicipital groove to take pressure off the shoulder [5], leading to significant relief of pain and regaining full or near full mobility of the shoulder. The success of the surgery relies largely on an effective bony incorporation (osteointegration) of the tendon graft, which is then expected to improve its pull-out tensile strength. However, there has been a relatively high failure rate that can be up to 30% [6]. Although a major cause for failure was related to other associated shoulder pathologies not addressed at the time of surgery [6], approximately one third of the failure was caused by inadequate fixation of the graft and the lack of bony integration of the graft [7], [8]. Management of a failed tenodesis and the associated shoulder pain may require revision surgeries, such as conversion of proximal biceps tenodesis to a subpectoral tenodesis [7]. Accordingly, an effective therapy that promotes osteointegration of the tendon graft would not only accelerate the healing time and expedite rehabilitation, but would also reduce the need for revision surgeries.

As in fracture repair, the tendon-to-bone healing involves three major phases of cellular actions: inflammation, repair, and remodeling [9]. The gap between the tendon and bone is initially filled with inflammatory cells. It is then gradually invaded by blood vessels, allowing cells to remove debris and for cells that form collagen to enter the gap. The formation of collagen and restoration of the bone-tendon interface progresses until the normal tendon insertion site is restored. The normal bony insertion site of the tendon exhibits four distinct histologic transitions: 1) type III collagen Sharpey-like fibers in the tendon, 2) uncalcified fibrocartilage, 3) calcified fibrocartilage, and 4) bone [10]. Tendon tissue heals very slowly and usually is healed by reactive scar formation. The lack of regeneration of normal tissue at the tendon-bone interface has been suggested to be due to insufficient growth factor production [11], inadequate mesenchymal stem cell (MSC) recruitment [12], and/or reduced mechanical load related to the decreased tendon-bone interface motion [13]. Thus, a number of protein- or gene therapy-based strategies that apply growth factors, primarily bone morphogenetic proteins (BMPs), have been attempted to accelerate tendon-to-bone healing in various animal models of tendon injuries, and have yielded varying degrees of limited success [14]–[18]. We have also recently attempted a lentiviral (LV)-based *BMP4 in vivo* gene transfer strategy to promote healing of biceps tenodesis, in which the LV-*BMP4* viral vector was administered directly into the bone-tendon interface inside the bony tunnel of a rat model of biceps tenodesis [19]. While this LV-*BMP4*-based *in vivo* gene transfer strategy markedly increased *de novo* bone formation on the bone surface of the bony tunnel, it did not enhance the bony reintegration (osteointegration) of the tendon graft. The LV-*BMP4 in vivo* gene transfer strategy did yield a small, but statistically significant, improvement in the return of the pull-out tensile strength of the tendon graft, presumably as the result of the bone formation effect of *BMP4* that traps or anchors the tendon graft onto the bony tunnel [19]. This study suggests that our *in vivo* gene transfer strategy is an effective means to deliver transgene to the tendon-bone interface within the bony tunnel, and it also indicates that the *BMP4* gene is not an optimal transgene because it does not promote osteointegration of the tendon graft.

The present study sought to evaluate whether the *COX2* gene would be an effective transgene to promote osteointegration of the tendon graft. Our rationale for using the *COX2* gene is threefold: first, despite the conflicting results of early studies with non-selective NSAIDs [20]–[22], recent studies have shown that selective NSAIDs for COX2 (e.g., parecoxib, celecoxib, valdecoxib) had detrimental effects on the tendon-to-bone healing and on the mechanical strength return and integrity of the healing tendon in several rat models [23]–[26]. Second, injury to primate flexor tendons in organ cultures increased PGE_2_ secretion *in vitro* [27] and treatment of human patellar tendon fibroblasts with PGE_2_ *in vitro* markedly reduced tendon fibroblast contraction [28], which is essential for scar tissue formation [29]. These findings suggest that COX2/PGE_2_ plays a suppressive role in the reactive scar formation. COX2/PGE_2_ has also been shown to have an important enhancing function during the proliferative phase of tendon healing [30]. Third, COX2 gene therapy promoted bony bridging of fracture gaps during fracture repair [31]. Because 1) osteointegration of tendon grafts and the bony bridging of the fracture gaps undergo similar histologic transition from cartilage to bone [10], and 2) the LV-*COX2 in vivo* gene transfer strategy accelerated the bony remodeling of the cartilage of the healing fracture callus in a mouse multiple tibial fractures model [32], we speculate that the *COX2 in vivo* gene transfer strategy could promote bony remodeling and osteointegration of the tendon graft. Accordingly, the primary objective of the present study was to evaluate the efficacy of the *in vivo COX2* gene transfer strategy on promoting osteointegration of the tendon graft and enhancing its mechanical pull-out strength using our rat model of biceps tenodesis [19].

## Results

### Characterization of normal healing after biceps tenodesis in the rat

Before we evaluated the effect of the LV-*COX2 in vivo* gene transfer strategy on the tendon-to-bone healing, we first characterized the time course of normal healing of biceps tenodesis without intervention in the rat. We qualitatively monitored the healing by scoring several parameters at the healing site at 3-, 5-, or 8-weeks post-surgery: cellularity (1, mild; 2, moderate; 3, marked), vascularity (1, mild; 2, moderate; 3, marked), fiber diameter (1, small, 2, medium; 3, large), cells parallelism (1, <25%; 2, 25–50%; 3, 50–75%), fiber binding to bone (1, continuity but not parallel; 2 continuity and in-growing; 3, ingrowing and tidemark), collagen birefringence (1, <25%; 2, 25–50%; 3, 50–75%), cartilage (metachromasia) (1, minimal; 2, mild; 3, moderate), and fibrocartilage (1, minimal; 2, mild, 2; moderate). The scoring was performed by a single investigator in a blinded fashion. Our assessment revealed no appreciable healing after 3 or 5 weeks. Mild to moderate levels of healing in several parameters were seen after 8 weeks (Table 1).

**Table 1:** Time-dependent changes in various cellular and structural parameters at the healing site of untreated biceps tenodesis in the rat.

Parameter	Three weeks post-surgery	Five weeks post-surgery	Eight weeks post-surgery
Cellularity*	3 (Marked)	2 (Moderate)	1 (Mild)
Vascularity	3 (Marked)	2 (Moderate)	1 (Mild)
Fiber diameter	1 (Small)	2 (Medium)	2.5 (Medium-large)
Cell parallelism	1 (<25%)	2 (25–50%)	3 (50–75%)
Fiber binding to bone	1 (Continuity but not parallel)	2 (Continuity and ingrowing)	3 (ingrown and tidemark)
Collagen birefringence	1 (<25%)	2 (25–50%)	3 (50–75%)
Cartilage (metachromasis)	1 (Minimal)	2 (Mild)	3 (Moderate)
Fibrocartilage	1 (Minimal)	2 (Mild)	3 (Moderate)

*Please see text for definition. Assessment was performed by an investigator (in 3–4 animals per time point) in a blinded fashion (i.e., without knowing the identity of the sample).

**Figure 1:**
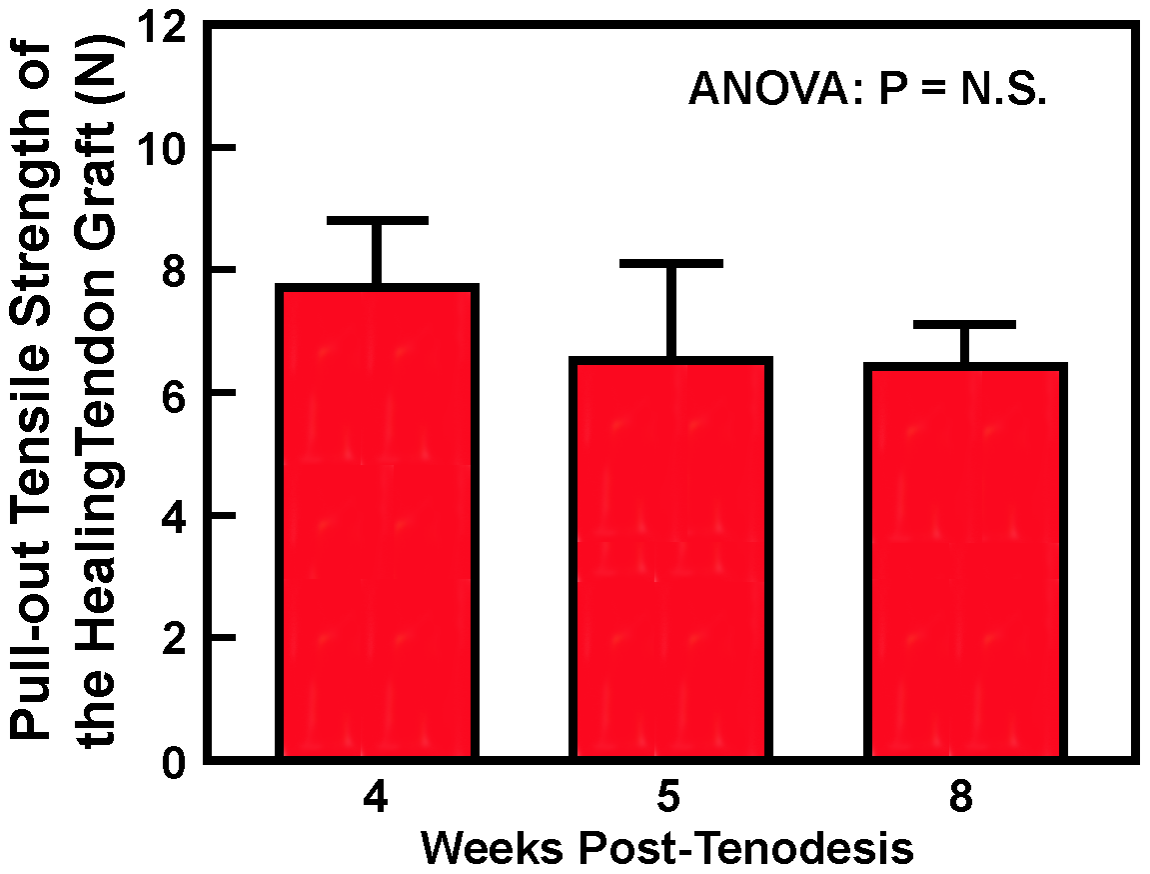
The pull-out tensile strength of untreated biceps grafts at 4, 5, or 8 weeks post-surgery. The pull-out tensile strength of untreated biceps tendon grafts (in Newtons) at each time point was determined as described previously (19). The average pull-out tensile strength of intact biceps tendons in these animals was 26.9 N. Results are shown as mean ±S.D. (n = 6 per time point).

We next evaluated the time-dependent effects of the healing on the return of the mechanical strength of the tendon graft by determining the pull-out tensile strength of the untreated grafts after 4, 5, and 8 weeks of the healing without intervention. No significant difference in the ultimate load of failure was noted at 4, 5, or 8 weeks, as the average pull-out tensile strength of the healing tendon graft at each test time point was between 6 and 7 Newtons (N) (Fig. 1). The average pull-out tensile strength of the intact biceps tendon of these rats was 26.9 N. Thus, the return of pull-out tensile strength for these healing grafts was only between 24% and 28%, indicating no improvement in the pull-out tensile strength of the tendon graft without intervention, even after 8 weeks of healing.

### The LV-*COX2 in vivo* gene transfer strategy stimulated *de novo* bone formation and neo-chondrogenesis at the tendon-bone interface of the bony tunnel

**Figure 2:**
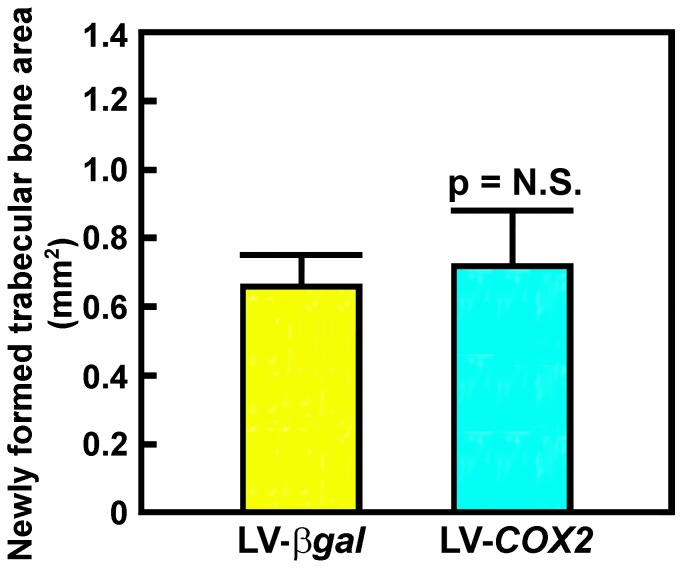
Quantification of newly formed trabecular bone area at the interface between the pin and bone surface in the bone tunnel of LV-*COX2*- or LV-*βgal*-treated rats after 5 weeks of healing. The area of the newly formed bone was measured with the Osteometric system. Results are shown as mean ±S.D. (n = 6 per test group).

**Figure 3:**
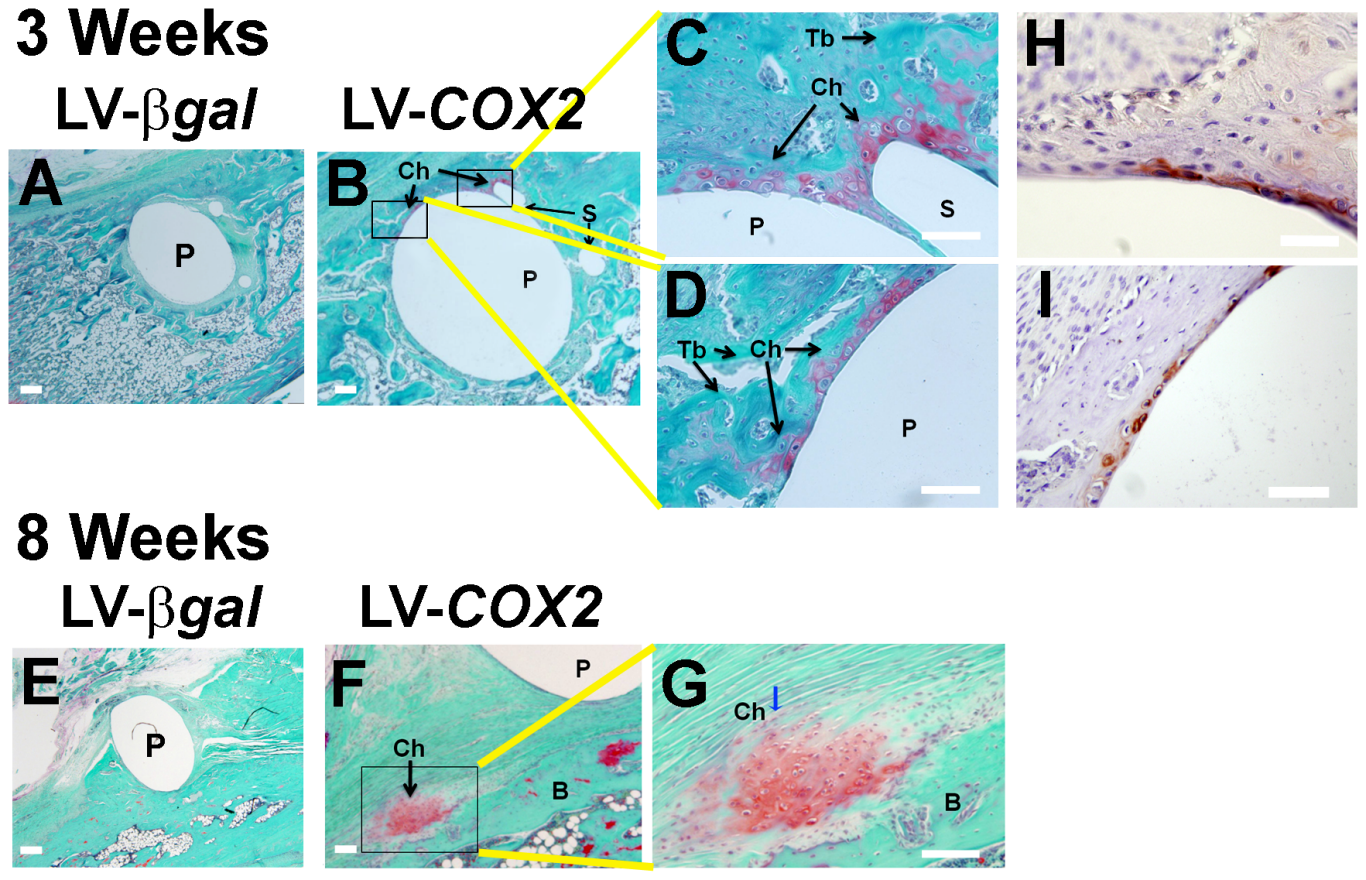
Histological staining of the biceps graft of rats treated with the control LV-*βgal* vector (A&E: 20X) or with the LV-*COX2* vector (B&F: 20X, C and D: 100X of the two boxed areas in B, G: 100X of the boxed area in F) for 3 weeks (A–D) and 8 weeks (E–G), respectively. The tenodesis humerus of a representative rat receiving the LV-*βgal* control vector (A&E) or the LV-*COX2* vector (B, C, D, F, and G) was de-mineralized and sectioned at an orientation perpendicular to the direction of the pin insertion. The slice corresponding to the pin-bone tunnel intersection was stained with light green and Safranin-Orange. Cartilage (chondrocytes) areas are stained red. S: suture hole; P: pin hole; Ch: cartilage; Tb: trabecular bone; and B: bone. The arrows in B show various foci of neo-cartilage formation at the interface between the tendon graft and the bone surface of the bony tunnel. The blue arrow in G shows the site of neo-cartilage formation where columns of chondrocytes were organized in parallel to the tendon fibers, a characteristic suggestive of being fibrocartilage. Panel H and I show two representative areas of the immunohistochemical staining for type II collagen (stained in brownish color) on a serial section (100X). Scale bars  = 100 µm.

Our previous study shows that direct administration of LV-*BMP4* vector into the tendon-bone interface of the bony tunnel significantly enhanced bone regeneration and neo-chondrogenesis at the tendon-bone interface inside the bony tunnel [19]. Thus, we assessed whether this *in vivo* gene transfer approach to deliver the LV-*COX2* vector to the tendon-bone interface would also promote *de novo* trabecular bone formation and neo-chondrogenesis at the bone-tendon interface after 5 weeks of treatment. Contrary to the LV-*BMP4 in vivo* gene transfer strategy that showed a 35% increase in trabecular bone formation at the tendon-bone interface after 5 weeks of treatment [19], the LV-*COX2* gene transfer strategy did not increase significant amounts of *de novo* trabecular bone formation at the tendon-bone interface (Fig. 2). The inability of the treatment to promote bone formation was probably not due to insufficient treatment time, since there was also no increase in *de novo* bone formation even after 8 weeks (data not shown). In contrast, staining with light green and Safranin-Orange for cartilage reveals the formation of numerous foci of neo-chondrogenesis at the tendon-bone interface (indicated by arrows in Fig. 3B) of the LV-*COX2*-treated biceps tenodesis at 3 weeks of healing. In addition the orientation of the cartilage within the foci of neo-chondrogenesis at the tendon-bone interface at 8 weeks after the LV-*COX2* administration was parallel to the tendon fibers (indicated by the arrow on Fig. 3G), a characteristic that is consistent to be fibrocartilage. However, similar to the effect produced by the LV-*BMP4* treatment [19], the foci of neo-cartilage formation induced by the LV-*COX2* treatment were also sparse and uneven. Consistent with this interpretation, immunostaining of type II collagen (a marker gene product of proliferative chondrocytes) on serial slices also showed similar sparse and uneven foci of immunostaining (Figs. 3H and I).

**Figure 4:**
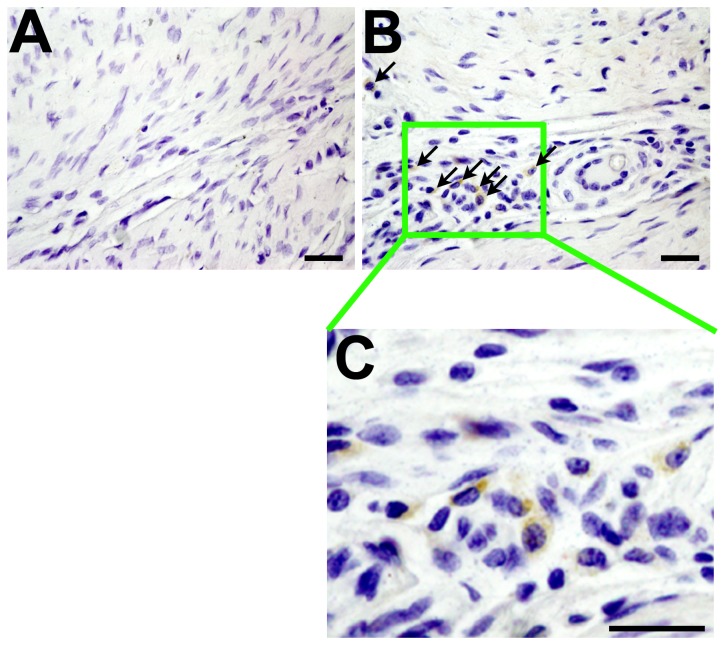
*In situ* hybridization analyses of human *COX2*-expressing cells at the tendon-bone interface of LV-*βgal*-treated control (A) or LV-*COX2*-treated (B and C) rat shoulders. The tenodesis humerus of LV-*βgal*-treated or LV-*COX2*-treated rats were harvested after three weeks of treatment and were each de-mineralized and sectioned at an orientation perpendicular to the direction of the pin insertion. The attachment site of the tendon is beyond the upper right hand corner of each panel. A mixture of three oligonucleotide probes for human *COX2* gene were applied to LV-*βgal* (control)-treated (A) or LV-*COX2*-treated (B and C) thin bone sections corresponding to the tendon-bone interface within the bony tunnel. Human *COX2*-expressing cells were seen only in the LV-*COX2*-treated (B), but not the LV-*βgal*-treated control sections (A). In panel B, arrows point to the human *COX2*-expressing cells. To assist identification of the human *COX2*-expressing cell loci, the area corresponding to the human *COX2*-expressing cells locus in panel B was enlarged 2.5-time and was shown in panel C. Scale bars  = 100 µm.

To evaluate whether the observed uneven distribution of foci of neo-chondrogenesis was related to the intrinsic difficulties associated with direct application of the viral vector that led to uneven transgene expression at the tendon-bone interface, we examined the distribution profile of the βgal marker gene expression (by histochemical staining) at three weeks at the tendon-bone interface inside the bony tunnel of four animals receiving the LV-*βgal* vector. The βgal transgene expression at the tendon-bone interface (data not shown) also showed similar uneven distribution profile as those of neo-chondrogenesis (Fig. 3 C&D) and type II collagen expression (Fig. 3 H&I). To further confirm that uneven distribution of foci of neo-chondrogenesis was related to uneven, focal expression of the human *COX2* transgene mRNA at the tendon-bone interface, we performed an *in situ* hybridization analysis for the human *COX2* transgene expression profile at the tendon-bone interface of LV-*COX2*- and LV-*βal*-treated tenodesis at three weeks post-treatment (Fig. 4). As expected, human COX2-expressing cells were seen only in LV-*COX2*-treated tenodesis (Fig. 4B and C), but not in LV-*βgal*-treated control shoulder (Fig. 4A). Similar to the distribution profile of neo-chondrogenesis (Fig. 3 C&D) and type II collagen (Fig. 3H&I) in LV-*COX2* treated shoulders, the distribution profile of human *COX2*-expressing cells on the LV-*COX2*-treated bone was also sparse and uneven. Together, these findings support the likely possibility that the observed uneven distribution of neo-chondrogenesis foci was in a large part a result of uneven expression of the transgene at the tendon-bone interface.

### The LV-*COX2 in vivo* gene transfer strategy promoted osteointegration of the tendon graft

**Figure 5:**
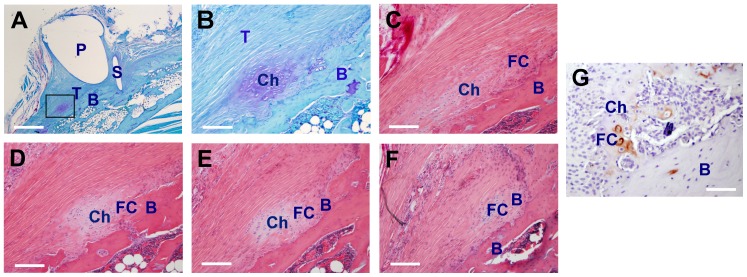
Histology of serial sections of the biceps tendon graft of a representative rat treated with LV-*COX2* for 8 weeks. The tenodesis shoulder receiving LV-*COX2* vector for 8 weeks was de-mineralized and serially sectioned (5 µm slides from the top) at an orientation perpendicular to the direction of the pin insertion. A and B: Slide 22 of areas around the bony tunnel stained with Toluidine blue (A at 20X, and B at 100 X of the boxed area of panel A); C-F are 100X of the boxed area of panel A in various serial sections (slides 16, 21, 25, and 30, respectively) and each was stained H&E. G: a serial section was stained immunohistochemically for type II collagen (stained in brownish color). P: pin hole; S: suture hole; T: tendon; Ch: cartilage; FC: fibrocartilage; and B: bone. The serial sections from panel C to F show that the tendon graft has undergone histological transition from cartilage to fibrocartilage and then to bone. Scale bar in A = 500 µm; and scale bars in B to G = 100 µm.

Toluidine blue staining of the LV-*COX2*-treated tenodesis site at 8 weeks indicates that the cartilage loci were surrounded by bony tissues (Figs. 5A&B). H&E staining of serial sections at the same site (Figs. 5C-F) provides clear histological evidence that the tendon graft gradually transited into cartilage-like tissue (fibrocartilage), which then transited into bony tissue and fused onto the existing cortical bone. Immunostaining for type II collagen on a serial section (Fig. 5G) shows that the area corresponding to the location of fibrocartilage was stained positively for type II collagen, supporting the contention that fibrocartilage was regenerated between the tendon graft and the bone surface of the bony tunnel. This gradual transition of tendon to cartilage-like tissue and then to bony tissue is similar to the histological transition of tendon tissue-to-fibrocartilage-to-bone seen at the normal insertion site of the biceps tendon at the gleno-humeral joint [19]. This would suggest that the onset of the osteointegration process may have occurred in the LV-*COX2*-treated tendon grafts after 8 weeks.

### The LV-*COX2 in vivo* gene transfer strategy increased angiogenesis at the tendon/bone interface of the healing tendon graft

Our investigation into the molecular and cellular mechanism of a similar LV-*COX2 in vivo* gene transfer strategy to promote bony union of fracture gaps in a mouse multiple fracture model strongly suggests that the *COX2* gene therapy promoted bony remodeling of callus cartilage, in part through an enhanced mesenchymal stem cell (MSC) recruitment and an increased *de novo* angiogenesis [32]. Because the tendon is relatively hypocellular and because the lack of sufficient MSC recruitment to the tendon-bone healing site has been implicated as a potential cause for the lack of regeneration of a normal tendon-bone insertion site [12], we next evaluated whether the LV-*COX2 in vivo* gene transfer strategy promoted osteointegration of the tendon graft also in part through an enhanced MSC recruitment and an increase in *de novo* angiogenesis. As an indirect assessment of MSC recruitment, we isolated total RNA in tissues at the bony tunnel of tenodesis shoulders (and corresponding tissues of the contralateral unoperated shoulders) of three rats each one week after receiving either the LV-*COX2* or the LV-*βgal* treatment. The mRNA expression levels of three MSC marker genes (i.e., Nestin, Podxl, and CD49f) were measured by qRT-PCR and normalized against corresponding level of cyclophilin (*Ppia*) mRNA, using primers specific for rat Nestin, Podx1, or CD49f, respectively. The LV-*COX2* treatment significantly increased Nestin mRNA (by ∼30-fold), Podxl mRNA (by ∼7-fold), and CD49f mRNA (by ∼15-fold) compared to the LV-*βgal*-treated tenodesis controls, suggesting that the LV-*COX2 in vivo* gene transfer strategy may also promote MSC recruitment to the healing site.

**Figure 6:**
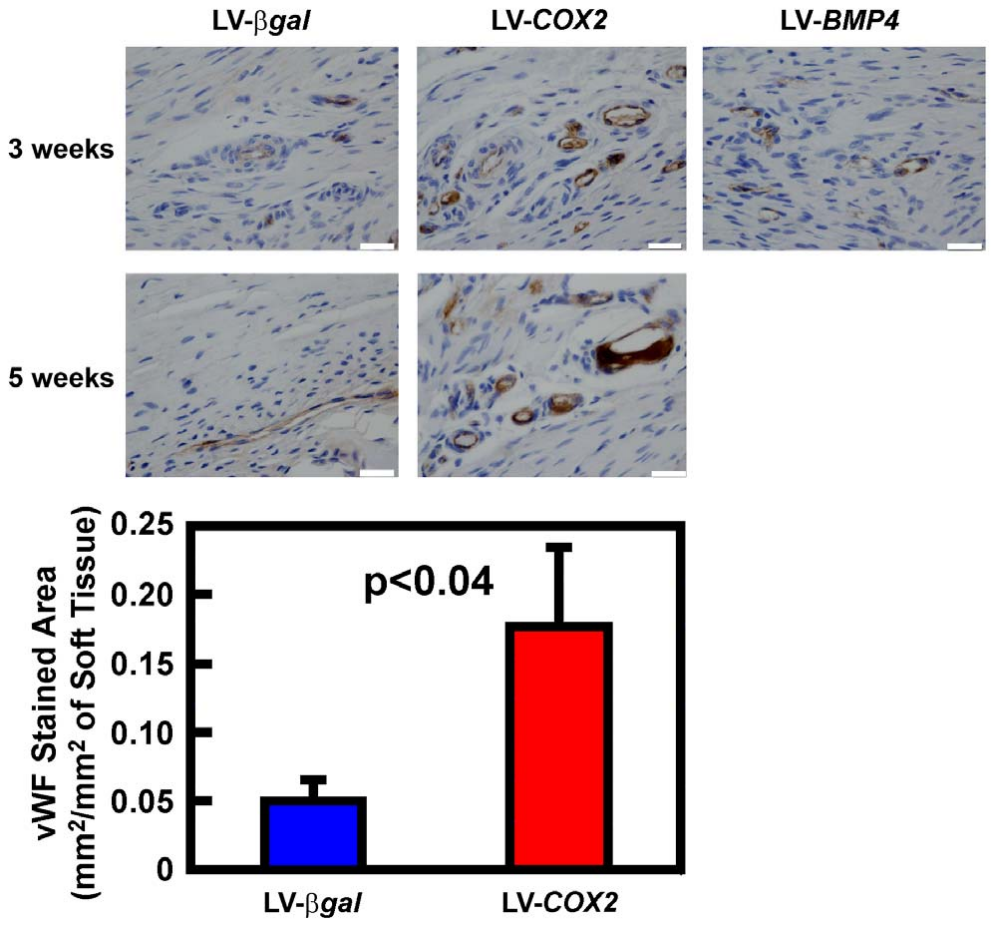
Comparison of the effect of the LV-*COX2 in vivo* gene transfer strategy with that of the LV-*BMP4 in vivo* gene transfer strategy on *de novo* angiogenesis at the tendon-bone interface within the bony tunnel. To identify blood vessels, thin sections of humerus containing the bony tunnel and the tendon graft of animals treated with LV-*COX2*, LV-*BMP4*, or LV-*βgal* control vector for either 3 weeks or 5 weeks were stained immunohistologically for vWF (brownish color) using a specific anti-rat-vWF antibody. Scale bars  = 100 µm. Bottom shows the quantification of the area stained for vWF per area soft tissue around the pinhole. Four individual samples each were examined for LV-*COX2* and LV-*βgal* (control) therapy at three weeks post-procedure. Three different areas of soft tissue were examined in each section. Statistical analysis was performed by two-tailed Student's t-Test.

To evaluate whether the *LV-COX2* gene transfer strategy for biceps tenodesis promotes angiogenesis, immunohistochemical staining for vWF (a blood vessel marker protein) was performed on thin sections of the proximal end of the humerus containing the bony tunnel from groups of three rats each, treated for 3 or 5 weeks with the LV-*COX2* or LV-*βgal* control vectors. A group of rats treated with the LV-BMP4 for 3 weeks was included for comparison. The LV-*COX2* gene transfer markedly increased the total vWF-stained blood vessel areas at the tendon-bone interface after 3 or 5 weeks compared the LV-*βgal*-treated controls, but the LV-*BMP4* treatment had no apparent effect after 3 weeks (Fig. 6). The vWF-satined blood vessel area per soft tissue unit area around the pinhole of groups of four animals each treated with either LV-*COX2 or* LV-*βgal* for three weeks were quantified at three randomly chosen areas using the ImagePro software (bottom panel of Fig. 6). The LV-*COX2* treatment, but not the LV-*BMP4* treatment (data not shown), significantly increased the blood vessel areas by more than three-fold. These findings indicate that the LV-*COX2* (but not LV-*BMP4*) gene transfer strategy stimulates angiogenesis at the tendon-bone interface.

### The LV-*COX2 in vivo* gene transfer strategy increased the pull-out tensile strength of the healing tendon graft

**Figure 7:**
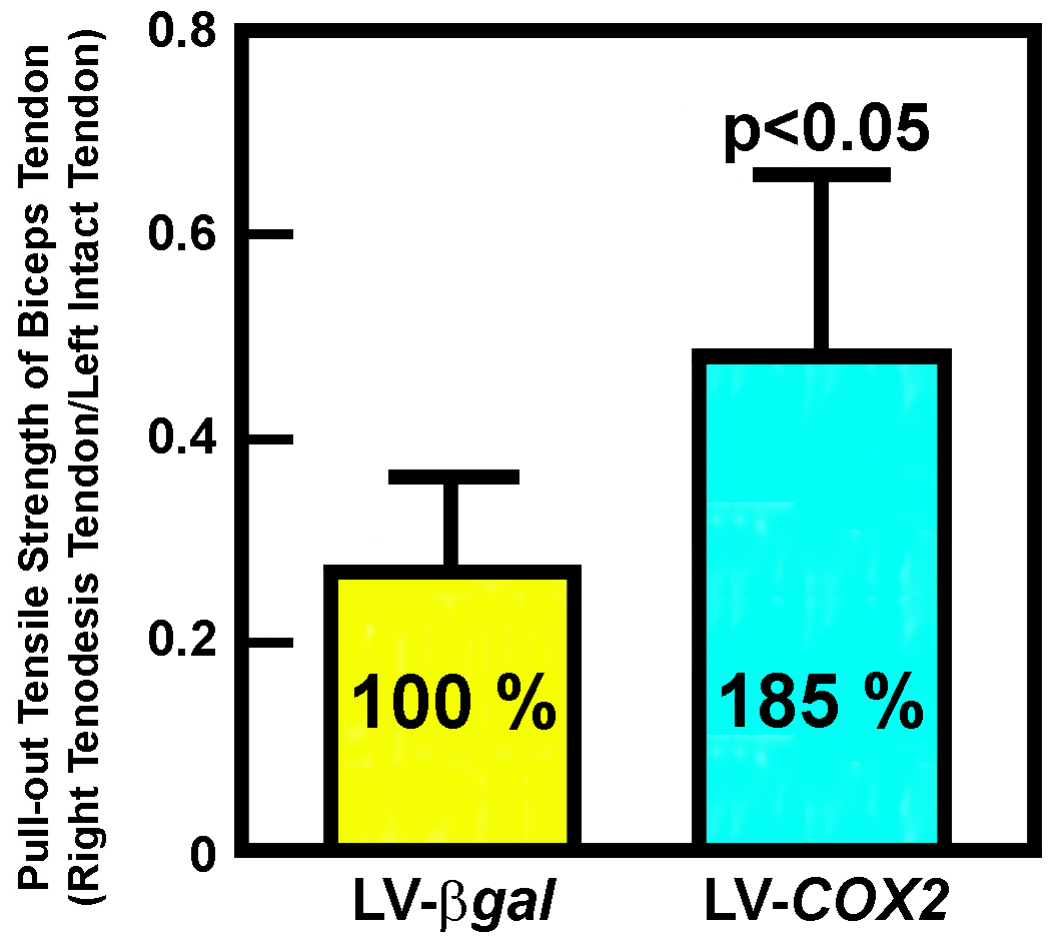
Comparison of the pull-out tensile strength of healing LHB tendon grafts of eight LV-Cox2-treated tenodesis shoulders with that of healing grafts of thirteen LV-*βgal*-treated control tenodesis shoulders after 5 weeks of healing. The return of pull-out tensile strength of the healing biceps tendon was shown as the relative ratio of the pull-out tensile strength of the operated right shoulders against the pull-out tensile strength of the intact biceps tendon of the left shoulder (i.e., right/left ratio). Results are shown as mean ±S.D. and statistical significance was determined with one-tailed Student's t-test.

To evaluate whether the enhancing effect of the LV-*COX2* treatment on osteointegration would result in an improvement in the tensile strength of the tendon graft, we measured the pull-out strength of biceps tendon grafts after 5 weeks of LV-*COX2*-treatment (Fig. 7). The *COX2* treatment significantly (p<0.05) increased the return of pull-out strength by 85% (from 0.26±0.11 to 0.48±0.16, p<0.05). We previously reported that under the same treatment conditions, the LV-*BMP4* treatment only marginally increased (by 29%) the return of the pull-out tensile strength of the tendon graft (from 0.26±0.11 to 0.34±0.09. p = 0.06) [19]. Thus, the increase in response to the LV-*COX2* treatment was two- to three-fold larger than that achieved by the LV-*BMP4* treatment.

## Discussion

The tendon-to-bone healing of untreated tendon grafts after tenodesis is poor (Table 1) and has often yielded little or no meaningful improvement in its pull-out tensile strength (Fig. 2). Accordingly, a number of growth factors-based protein, cell, or gene transfer therapeutic strategies have been attempted in the past to improve upon the tendon-to-bone healing in several tendon surgery models; many of which resulted in substantial amounts of cartilage and bone formation at the tendon-bone interface. Unfortunately, their beneficial effect on the pull-out tensile strength of the tendon graft, which is the ultimate clinical objective, was usually unimpressive [14]–[18]. We have also recently reported that the LV-*BMP4 in vivo* gene transfer strategy did not promote osteointegration and produced only marginal improvement in the return of pull-out tensile strength of the graft, despite large increases in bone and cartilage formation at the tendon-bone interface [19]. The lack of a significant benefit of these therapies on the return of pull-out strength is presumably due to the lack of enhancing effects on osteointegration of the tendon graft.

In this study, we have obtained compelling histological evidence that direct application of the LV-*COX2* vector to the tendon-bone interface at the time of the tenodesis surgery induced osteointegration of the tendon graft. This conclusion is based on our histological analyses of serial sections of tissues at the tendon graft-bone junction, which have offered unambiguous evidence for the gradual transition of the tendon graft into fibrocartilage and the subsequent conversion of fibrocartilage into bony tissues, which then connected to the existing bone within the bony tunnel (Fig. 5). More importantly, this *in vivo* gene transfer strategy yielded substantial biomechanical benefits, as the return of pull-out tensile strength of the LV-*COX2*-treated tendon grafts was improved by an average of 85% (p<0.05) over that of the control LV-*βgal*-treated tendons after only 5 weeks of healing (Fig. 7). This increase corresponds to the return of its pull-out tensile strength approaching an average of ∼50% of the pull-out tensile strength of the corresponding contralateral intact tendons. Recent studies have suggested that direct application of recombinant PTH [33] or of MSCs genetically modified to overexpressing *MT1-MMP* [34] or scleraxis [35] into the bony tunnel each may promote tendon-to-bone healing in a rat supraspinatus tendon repair model. However, the assumption was based primarily on the findings that each treatment produced significantly more mineralized fibrocartilage at the tendon-bone interface of the treated animals than corresponding control animals. There was no compelling histological evidence that the osteointegration of the tendon graft had indeed occurred. The fact that our recent LV-*BMP4 in vivo* gene transfer strategy, which also increased the amounts of fibrocartilage and new bone formation at the tendon-bone interface in the rat model of biceps tenodesis [19], but did not promote osteointegration of the tendon graft (i.e., lack of the four histologic transitions from tendon to bone at 8 weeks of the LV-*BMP4* treatment), suggests that one cannot simply assume, just because a therapy (such as the PTH [33], MSC/*MT1-MMP* [34], or MSC/scleraxis [35] treatments) is able to promote fibrocartilage and bone formation at the tendon-bone junction, that this therapy will also promote osteointegration. Consequently, this LV-*COX2 in vivo* gene therapy appears to be the only strategy that has clearly been shown to induce osteointegration of the tendon graft.

Similar to the LV-*BMP4* treatment, the LV-*COX2 in vivo* gene transfer strategy also stimulated neo-cartilage (fibrocartilage) formation at the tendon-bone junction (Fig. 3); but unlike the LV-*BMP4* treatment [19], the LV-*COX2* treatment strategy did not induce new bone formation at the tendon-bone junction (Fig. 2). However, the enhancing effect of the LV-*BMP4 in vivo* gene transfer strategy on the return of pull-out tensile strength of the tendon graft was only marginal (29.5±11.8% improvement, p = 0.066) after 5 weeks [19], which was approximately 3-fold less than that of the LV-*COX2* strategy (Fig. 7). That the LV-*BMP4* gene therapy produced only small improvement in the return of pull-out integrity of the tendon graft despite the large increase in new bone formation is consistent with several previous studies that showed small improvement in the mechanical strength of the tendon graft treated with the BMP protein [36], [37] or gene therapy [14], [15], [18]. We speculate that the amounts of new bone formed at the tendon-bone interface in response to the BMP4 therapy may be sufficient to wrap around the tendon graft, which then provides anchoring sites such that slightly more force is required to pull the graft out of the bone socket [19]. The much greater improvement in the return of the pull-out tensile strength seen in LV-*COX2*-treated tendon grafts compared to that of the LV-*BMP4*-treated grafts is most probably due to the *COX2*-induced osteointegration, rather than new cartilage or bone formation.

The cellular mechanism by which the *COX2*-based gene transfer strategy promotes osteointegration is unclear at this time. However, our findings that the LV-*COX2*, but not the LV-*BMP4*, *in vivo* gene transfer strategy enhanced neo-angiogenesis at the tendon-bone junction and osteointegration (Fig. 6) could provide mechanistic insights and also suggest that *COX2*-induced osteointegration may involve *COX2*-mediated upregulation of neo-angiogenesis at the tendon-bone junction. This interpretation is consistent with previous studies showing that angiogenesis is essential for the tendon-to-bone healing [38], [39]. We should note that a similar LV-*COX2 in vivo* gene transfer strategy also promoted bony bridging of fracture gaps of multiple tibial fractures and that angiogenesis plays a crucial role in the COX2-induced bony bridging of the fracture gap [31], [32]. Accordingly, we showed that the *COX2 in vivo* gene transfer strategy significantly enhanced angiogenesis between day 14 to day 21 post-fracture, which immediately preceded the remodeling of cartilaginous callus to bony tissue [31], [32], and that blocking this angiogenesis process with an inhibitor (endostatin) completely abrogated the *COX2*-mediated bony remodeling of the cartilaginous callus and the bony bridging of the fracture gap. The various histologic phases of fibrocartilage-to-bone transition seen in osteointegration [10] are reminiscent to those of cartilage-to-bone remodeling in endochondral bone formation during fracture repair [40]. It is conceivable that enhanced angiogenesis at the tendon-bone interface (or at the fracture callus) would play an essential role in the *COX2*-mediated induction of the transition of tendon tissues to fibrocartilage and the transition of mineralized fibrocartilage into bony tissues, and in the remodeling of the newly formed bone to fused into the existing cortical bone within the bony tunnel to complete the osteointegration process. In support of our tentative conclusion that enhanced angiogenesis is essential for osteointegration, the LV-*BMP4 in vivo* gene transfer strategy, which did not promote neo-angiogenesis at the tendon-bone interface (Fig. 6), also did not induce osteointegration. Similarly, the *BMP4 in vivo* gene transfer strategy, which had no enhancing effect on neo-angiogenesis in the fracture callus, also did not promote bony union of the fracture gap in a rat femoral fracture model [41].

Our recent studies with the *COX2*-based gene therapy of fracture healing have suggested that the early fracture healing phase appears to involve recruitment of MSCs to the fracture site through increased local SDF1 production, and that the MSC recruitment is enhanced by the *COX2* gene therapy [32]. In this regard, the tendon tissue is relatively hypocellular, and insufficient MSC recruitment to the healing interface has been implicated as a potential cause for the lack of regeneration of a normal tendon-bone insertion site [12]. Our measurements of relative gene expression levels of several marker genes of MSC (i.e., Nestin, Podx1, or CD49f) at the tendon-bone interface within the bony tunnel one week after the treatment have indicated that the LV-*COX2 in vivo* gene transfer strategy significantly increased the expression levels of these MSC marker genes, suggesting that the *COX2* gene therapy may have also promoted MSC recruitment during the early phase of the tendon-to-bone healing. Our future studies will confirm this interesting, but tentative, conclusion.

We should note that the current LV-*COX2 in vivo* gene transfer strategy has a significant limitation; that is, the foci of the LV-*COX2*-induced osteointegration sites (i.e. neo-cartilage formation sites) at the tendon-bone junction were rather spotty and uneven (Fig. 3). It could be due to the intrinsic difficulties associated with direct application of the viral vector to the tendon-bone interface (Fig. 4). Accordingly, despite extreme care we found it very difficult to apply the same amounts of the viral vector evenly across the entire tendon-bone junction. As a result, the resulting spotty and uneven foci of osteointegration yielded variable biological effects. For example, while the average return of the pull-out tensile strength of the LV-*COX2*-treated tendon graft was 49±16% after 5 weeks of the treatment (Fig. 7), it ranged from 34% to 76%. It is conceivable that the beneficial effect of the LV-*COX2* gene transfer strategy would be significantly much better and more consistent if we could develop a viral vector application strategy that would yield more uniform osteointegration. Accordingly, our laboratory is currently working on several strategies to improve on the target delivery of the viral vector to produce more uniform transduction of cells at and around the tendon-bone interface within the bony tunnel.

In summary, we have demonstrated for the first time that direct application of LV-*COX2* vector to the tendon-bone interface within the bony tunnel at the time of biceps tenodesis was able to promote osteointegration of the tendon graft, which resulted in marked improvement in the return of the pull-out strength of the tendon graft in a reasonable time frame. These exciting findings raise the intriguing possibility that this LV-*COX2 in vivo* gene transfer strategy may be developed into an effective therapy to accelerate the tendon-to-bone healing of tendon graft. Currently, the surgically repaired tendons must be protected for at least 6 weeks followed by a more generally guarded period for the next 3 to 6 months. An improvement in the tendon-to-bone healing by this *COX2*-based therapy should yield healing tendon grafts with better return of their pull-out strength. This could then shorten the rehabilitation process and may even allow more aggressive rehabilitation techniques. Patients undergoing tendon or ligament reconstruction surgeries would benefit from being able to rapidly progress into a more active and challenging phase of rehab to avoid stiffness, weakness and prolonged immobilization.

## Materials and Methods

### The rat model of biceps tenodesis

All animal protocols have been reviewed and approved by the Institutional Animal Care and Use Committee of the Jerry L. Pettis Memorial VA Medical Center. The rat model of biceps tenodesis used in this study has been described previously [19]. In this surgical model, 12-week-old male Fischer 344 rats (Charles River Laboratory, Wilmington, MA, USA) were placed under general anesthesia with isoflurane. The right forelimb was prepared, draped, and a longitudinal incision was made from the neck to the elbow to expose the shoulder. The cephalic vein was cauterized, and the anterolateral deltoid was released from the acromion and split down the raphe of the anterior and middle thirds. The long head of the biceps tendon was identified and followed cephalad to the intertubercle groove. The transverse intertubercle ligament was released. The tendon was separated from the muscle to its origin on the glenoid and released. It was whip-stitched with 5–0 prolene to produce a traction suture. A bony tunnel was drilled in the mid-groove using a 1.14-mm diameter Kirschner wire and a straight needle used to pass the traction suture through both cortices of the tunnel. At this time, the LV-*COX2* vector or LV-*βgal* control vector was applied directly onto the end of the tendon graft and into the bony tunnel. The tendon end was pulled into the bone tunnel. A poly L-lactide bioabsorbable ‘SmartPin’ (diameter 1.1 mm; catalog #121110; ConMed Linvatec, Largo, FL, USA) [also coated with the viral vector] was press-fitted into the bony tunnel as interference fixation. The deltoid was re-approximated with a 4v0 PDS absorbable suture and the skin was closed in similar fashion. Buprenorphine was administered subcutaneously for postoperative analgesia, and the rat was allowed unrestricted mobility during recovery. No functional deficits in the animal were noted, and the animal did not favor the non-operated limb over the operated limb or had reduced physical activities during the healing period.

### *In vivo* administration of LV-based vectors to the tendon graft–bone interface inside the bony tunnel

**Figure 8:**
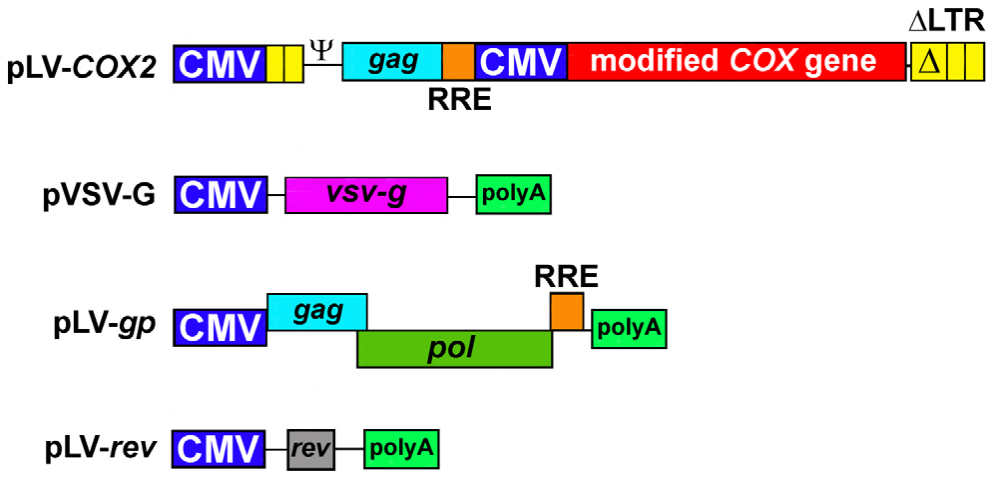
A schematic representation of the structure of expression plasmids used to produce the third-generation self-inactivating LV-*COX2* vector. The transfer expression construct (pLV-*COX2* plasmid) used the cytomegalovirus (CMV) promoter to drive expression of a human *COX2* gene that had been modified by removing the 3′-untranslated region (UTR) that contains multiple copies of the AUUA-rich element to increase the mRNA stability and also by replacing the native Kozak sequence with an enhanced Kozak sequence to improve protein translation (22). The expression of *gag*, *pol*, and *RRE* lentiviral genes on the pLV-GP plasmid, the *Rev* gene on the pLV-Rev plasmid, and the heterologus VSV-G envelope gene on the pVSV-G plasmid was driven by the CMV promoter. The transfer construct did not contain the wild-type copy of the LV LTR promoter (thereby self-inactivating). The 5′-LTR was chimeric, containing a CMV promoter replacing the U3 region to rescue transcriptional dependence on the *tat* lentiviral gene. The 3′-LTR (ΔLTR) contained a deletion through the U3 region that renders it transcriptionally inactive.

The LV-based vector expressing a modified human *COX2* gene (or the *βgal* marker gene) was produced by transient co-transfection of 293T cells with the four plasmid system (Fig. 8) as described previously [41]. In this modified human *COX2* gene, the 3'-UTR region (with exception of 14 nucleotides) that contains multiple copies of the AUUA-rich mRNA-decay element was deleted to increase the stability of the *COX2* mRNA. The native Kozak sequence of the *COX2* mRNA was also replaced with an optimized Kozak sequence (TCCACCATGG) to enhance the efficiency of COX2 protein translation [31]. During the viral administration procedure, a total of 3.75×10^7^ transforming units of LV-*COX2* or LV-*βgal* control vector (in a total volume of 30 µl) were applied carefully to the end of the tendon graft, on the surface of the pin, and into the bone tunnel immediately before insertion of the tendon graft.

The effectiveness of *in vivo* transduction of cells at the tendon–bone junction and inside the bony tunnel was confirmed by βgal expression in the LV-*βgal* transduced shoulder 3 weeks after the viral vector administration (data not shown). A group of five to eight animals per treatment group were sacrificed after 3 or 8 weeks of healing, the proximal humerus of the treated shoulder was harvested and the healing site (i.e. the bony tunnel) subjected to histological analyses. Another group of eight animals per treatment group were sacrificed after 5 weeks of healing to assess the pull-out tensile strength of the tendon graft.

### Histology

Tendon-to-bone healing was evaluated by the histology of the biceps tendon structures at the joint insertion site of the operated shoulder (compared with each respective contralateral, unoperated shoulder). After euthanasia, humeri were collected, fixed in 10% formalin for 24–48 h and decalcified in 14% EDTA in phosphate-buffered saline (pH 7.0) for 3 weeks. Bones were dehydrated in ethanol, embedded in paraffin and the proximal end longitudinally sectioned. Some samples were orientated in such a way that the pin was sectioned in the cross-sectional direction; and other samples were orientated so that the pin was sectioned longitudinally. The sections were often not completely intact because the pin was brittle and pieces of the pin disrupted the tissue. Sections were stained for hematoxylin and eosin (H&E), safranin orange, and toluidine blue.

Neo-angiogenesis was determined by immunohistological staining of von Wilebrand Factor (vWF) (as described in [31], [32]) on sections at the site corresponding to the tendon-bone interface within the bony tunnel at 3 weeks or 5 weeks after viral administration. Quantification was performed by a blinded observer using the Image Pro software, version 4.0 (Media Cybernetics, Bethesda, MD), which measured the area stained for vWF in mm^2^ per soft tissue area around the pinhole in mm^2^. Bone samples of 4 individual animals per treatment group were examined for LV-*COX2* and LV-βgal (control) therapy at three weeks post-procedure. Three different (randomly selected) areas of soft tissue were examined in each section with four animals per treatment group.

For type II collagen immunohistochemical staining, paraffin sections were de-parafinized, treated with hyaluronidase (0.01 g/µl) at 37°C to expose the collagen, and then treated with Rodent Block R (Biocare Medical, Concord, CA, USA) to minimize background staining. Sections were treated for 1 hour at room temperature with a monoclonal mouse anti-rat type II collagen antibody (Cat. # CIIC1, Developmental Studies Hybridoma Bank, University of Iowa, IA, USA). The antibody was detected by incubating the sections for 25 min at room temperature with BioCare's mouse-on-rat horse radish peroxidase (HRP) polymer kit.

### *In situ* hybridization analysis of human *COX2* gene expression

Because of the homology between the human *COX2* transgene and the endogenous murine *Cox2* gene, oligonucleotide probes were used. The oligonucleotides were 28-mer and 29-mer sequences designed to the human *COX2* gene (NM_011198.3) with the less than 65% homology to the murine *Cox2* gene. Three oligonucleotide sequences corresponding to exons 5 (647-620), 8 (1308–1280) and 9 (1441–1414) of the human *COX2* gene were gel-purified and end-labeled with biotin-14-dATP using terminal deoxyribonucleotide transferase (Invitrogen, Grand Island, NY). The three oligonucleotides were combined for use in the hybridization. A mixture of the complementary sequence of the three oligonucleotide probes (i.e., opposite sense of these sequences) served as a negative control probe.

Tissues were formalin-fixed, paraffin-embedded and sagittal sectioned. The human *COX2* oligonucleotide probe mixture was hybridized to sections that had undergone LV-*COX2* gene transfer treatment or the *LV-βgal* gene transfer control, and were harvested at three weeks post-procedure. Hybridization was performed at 37°C in 40% formamide and the final wash stringency was performed in 1X SSPE at room temperature. Hybridization was detected using a streptavidin-horse radish peroxidase (Vector Labs, Burlingame, CA) and 3,3′ diaminobenzidine (Betazoid DAB, Biocare, Concord, CA). Sections were counterstained with hematoxylin. Photomicrographs were obtained using an Olympus BX60 microscope and DP72 camera (Olympus America, Melville, NY).

### Pull-out tensile strength biomechanical test for rat biceps tendons

Our recently developed tensile pull-out strength mechanical testing method for rat biceps tendon was used to assess the return of pull-out tensile strength of the healing graft [19]. Briefly, tensile pull-out strength mechanical testing of the tendon graft was performed using an Instron 8800 servohydraulic tester (Instron Corp., Canton, MA, USA) and Wavemaker software (Editor 7.1.0, Instron Corp., Canton MA, USA) adapted to accommodate the small rat biceps tendons and the humerus. Both forelimbs were removed, and the surrounding tissues were dissected away from the biceps muscle, tendon and the humerus bone (or scapula for intact shoulder) to which that biceps tendon graft is reattached. The apparatus that connected the tendon to the Instron for testing was adapted to accommodate the operated or intact shoulders. The humerus of the operated shoulder was horizontally cradled in a Senn retractor, which was suspended from the Instron actuator. For un-operated intact shoulders, the scapula was directly clamped with an Allis clamp, which was then suspended from the Instron actuator. The tendon and muscle were allowed to hang vertically from the apparatus. The muscle was then cut at its junction with the remaining tendon grasped underneath the Senn retractor with a needle holder whose jaws had been cooled in liquid nitrogen to enhance their grip on the tissues. To secure the muscle/tendon unit to the frozen jaws of the retractor and to minimize frozen-related breakage of the muscle/tendon unit at the grasping site, several loops of suture line were placed at the tendon/muscle junction that allowed the frozen jaws of the retractor to grasp tightly onto the tendon/muscle unit. The handle of the needle holder was attached to the load cell. The tendon was pulled at constant force at 30 mm/min at a 90^o^ angle until it failed at the insertion site. The strength of this insertion in response to therapy was established as the maximum force at which it failed (ultimate load to failure). The load divided by the displacement indicated its stiffness. We have shown previously that the opposite orientation of the left vs. the right biceps tendon in relationship to the glenohumeral joint did not affect the tensile pull-out strength measurements [19].

### Statistical analysis

Statistical significance of differences was performed using a one-tailed, two-sample with equal variances, independent Student's t-test or a one-tailed, nonparametric Mann-Whitney U-test. The use of one-tailed statistical tests contains a pre-emptive bias in ignoring the possibility that the *COX2* would cause a reduction in tendon repair. p<0.05 was considered statistically significant. All data are reported as the mean ±SEM.
